# Somatic Mutations Profile of a Young Patient With Metastatic Urothelial Carcinoma Reveals Mutations in Genes Involved in Ion Channels

**DOI:** 10.3389/fonc.2019.00435

**Published:** 2019-05-29

**Authors:** Jyoti Sharma, Barnali Deb, Irene A. George, Shruthi Kapil, Karunakaran Coral, Nandita Kakkar, Smita Pattanaik, Arup Kumar Mandal, Ravimohan S. Mavuduru, Prashant Kumar

**Affiliations:** ^1^Institute of Bioinformatics, International Technology Park, Bangalore, India; ^2^Manipal Academy of Higher Education (MAHE), Manipal, India; ^3^MedGenome Labs Ltd., Bangalore, India; ^4^Department of Histopathology, Post Graduate Institute of Medical Education and Research, Chandigarh, India; ^5^Department of Pharmacology, Post Graduate Institute of Medical Education and Research, Chandigarh, India; ^6^Department of Urology, Post Graduate Institute of Medical Education and Research, Chandigarh, India

**Keywords:** NGS, bladder carcinoma, altered pathways, drugs, therapy

## Abstract

**Background:** Urothelial carcinoma is the most common malignancy of the bladder and is primarily considered as a disease of the elderly. Studies that address bladder tumor occurrence in young age groups are rare.

**Case Presentation:** A 19-year-old male presented with a gross total painless hematuria. A histology after biopsy revealed a high-grade transitional cell carcinoma with lymph node metastasis. The patient succumbed to the disease on day 72 of the treatment. Here, we used whole-exome sequencing of a paired tumor-normal sample to identify the somatic mutations and the possible targets of treatment.

**Result:** We predicted eight potential driver mutations (*TP53* p.V157L, *RB1* c.1498+1G>T, *MED23* p.L1127P, *CTNND1* p.S713C, *NSD1* p.P2212A, *MED17* p.G556V, *DPYD* p.Q814K, and *SPEN* p.S1078^*^). In addition, we predicted deleterious mutations in genes involved in the ion channels (*CACNA1S* p.E1581K, *CACNG1* p.P71T, *CACNG8* p.G404W, *GRIN2B* p.A1096T, *KCNC1* p.G16V, *KCNH4* p.E874K, *KCNK9* p.R131S, *P2RX7* p.A296D, and *SCN8A* p.R558H).

**Conclusions:** Most likely, mutations in genes involved in ion channels may be responsible for the aggressive behavior of a tumor. Ion channels are the second largest class of drug targets, and may thus serve as a putative potential therapeutic target in advanced stage urothelial carcinoma.

## Background

Urothelial carcinoma (UC) originates in the inner lining of the bladder epithelium and accounts for more than 90% of bladder cancer (1). Management of this cancer largely depends on prevention of progression and early identification of patients in non-muscle invasive bladder cancer (NMIBC) stage. Low-risk NMIBCs are likely to develop and progress to high-risk muscle invasive bladder cancer (MIBC). About 75% of the cases are NMIBC which tend to reoccur frequently (5–25%) and progress to the more aggressive MIBC (2). The median age for the diagnosis of urothelial carcinoma is approximately 69 years in males and 71 years in females (3). It rarely occurs in young adults (< 20 years of age) with a total incidence of < 200 cases worldwide by 2017 (4). UC in these rare cases are pathologically unique and have molecular features with very few genetic and epigenetic events reported (5). A list of standard drugs is routinely used to treat UC. Systemic treatment for this cancer has been restricted to cisplatin-based chemotherapy with negligible advancement over the past decades (6). Regardless of the treatment by transurethral resection combined with intravesical chemotherapy, more than 50% recurrence has been observed, and eventually 10–20% of these tumors progress to muscle invasive stage (7). Molecular studies in young adults would identify several oncogenic targets that could hold the promise for therapy. Whole-exome sequencing has not been carried out in the reported pediatric cases (8–14) and would aid in the identification of such targets. Here, we used whole-exome sequencing to identify somatic mutations and possible targets for treatment of a 19-year-old male patient with metastatic urothelial transitional cell carcinoma.

## Case Presentation

**Figure 1:**
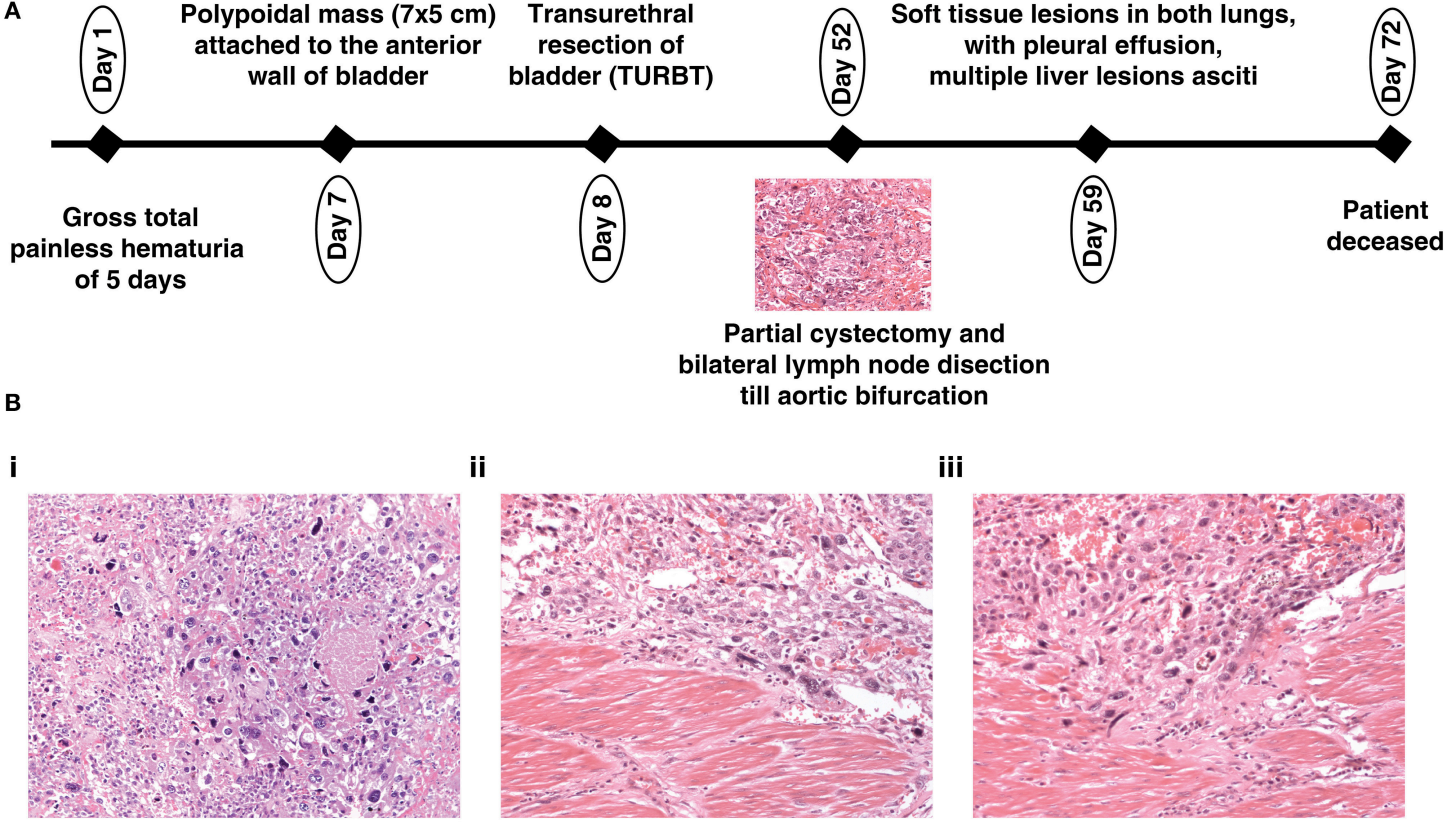
**(A)** Depiction of the history of a 19-year-old patient with urothelial carcinoma and **(B)** (20X magnification) Hematoxylin and Eosin stained section (i) showing a high-grade urothelial carcinoma with plenty of large pleomorphic cells; (ii,iii) showing a high-grade urothelial carcinoma infiltrating the detrusor muscle.

A 19-year-old male presented with gross total painless hematuria of a 5 days duration. There was no past family history of cancer. General physical examination and systemic examination were normal. Blood workup showed anemia. Renal function and liver functions were within normal limits. An ultrasound showed a polypoidal mass attached to the anterior wall of the bladder of 7 × 5 cm in size, which was further confirmed by a contrast enhanced CT scan (CECT) of abdomen. There was no evidence of lymph node or visceral metastasis. Transurethral resection of the bladder mass was performed. The upper gastrointestinal tract (GI) and lower GI endoscopy was within normal limits. A bone scan did not show any skeletal metastasis. Thereafter, the patient underwent robot assisted partial cystectomy and bilateral lymph node dissection till aortic bifurcation. The histopathology was suggestive of a high-grade urothelial carcinoma with six out of seven nodes showing metastasis. Post operatively, the patient developed fever and intestinal obstruction, initially managed conservatively, however, the patient did not show improvement. A repeat CECT abdomen was done which showed soft tissue lesions in both lungs, with pleural effusion, multiple liver lesions and ascites, suggestive of disseminated metastasis. The patient's general condition deteriorated, and he subsequently succumbed to his disease. An overview of the medical disease history is illustrated in a timeline (Figure 1A). Photomicrographs of the tumor from the urinary bladder showed a high-grade urothelial carcinoma with plenty of large pleomorphic cells and infiltrating the detrusor muscle (Figure 1B).

Whole-exome sequencing (WES) analysis of the paired tumor-normal sample from the patient was performed. A detailed description of the sequencing methods is provided in the Supplementary Material. WES data analysis revealed 558 exonic somatic mutations, of which 360 missense, 26 nonsense, 30 frameshift deletions/ insertions and, also 10 splice site mutations were annotated (Supplementary Figure 1). Thirty mutations are reported in COSMIC database (15) including in genes, such as *TP53, ABL1, ARID5B*, and *P2RX7* (Supplementary Table 1). In addition, using Cancer Genome Interpreter (16), we predicted eight potential driver mutations among all the somatic mutations detected in this rare tumor. These predicted driver mutations including loss-of-function mutations in *TP53, RB1, MED23, CTNND1* and activating mutations in *NSD1*and *MED17* (Table 1). The *TP53* p.V157L a known oncogenic mutation was identified as a recurrent hotspot in various cancer types (17). *RB1* is involved in the regulation of the cell cycle checkpoint and DNA damage response. The *RB1* c.1498+1G>T alteration is likely oncogenic. Mutations in *RB1* is associated with poor overall survival in patients with urothelial carcinoma (18). Domain structures of these genes highlighting the predicted deleterious mutations were generated using MutationMapper (Supplementary Figure 2).

**Table 1:** List of predicted somatic driver mutations in this patient.

**Gene**	**Chr: position**	**Ref allele**	**Mut allele**	**Codon change**	**Protein change**	**Variant type**
*TP53*	Chr17: 7578461	C	G	c.469G>C	p.V157L	Missense
*RB1*	Chr13: 48954378	G	T	c.1498+1G>T	−	SpliceDonorSNV
*MED23*	Chr6: 131914164	A	G	c.3380T>C	p.L1127P	Missense
*CTNND1*	Chr11: 57575908	C	G	c.2138C>G	p.S713C	Missense
*NSD1*	Chr5: 176721003	C	G	c.6634C>G	p.P2212A	Missense
*MED17*	Chr11: 93542965	G	T	c.1667G>T	p.G556V	Missense
*DPYD*	Chr1: 97700410	G	T	c.2440C>A	p.Q814K	Missense
*SPEN*	Chr1: 16255968	C	G	c.3233C>G	p.S1078^*^	Nonsense

Given that the above predicted driver mutations are in the genes that are limited to already known/predicted cancer driver genes, we carried out a network analysis of 347 genes that harbor a missense mutation using the STRING database. An analysis of the enriched interaction network was performed against the whole genome genes and the enrichment of ion channel pathways was identified (Supplementary Figure 3). Ion channels play a pivotal role in regulating self-sufficiency in growth, insensitivity to anti-growth signals, evasion of apoptosis, limitless replication potential, sustained angiogenesis, tissue invasion and metastasis (19, 20). We identified somatic alterations in 22 genes involved in the ion channels. Table 2 shows the list of seventeen missense and one frameshift insertion somatic mutations in genes involved in the ion channels. The human genome encodes approximately 328 ion channel genes^1^ (Supplementary Table 2A). Mutated genes in this patient belong to 11 groups of ion channels (Supplementary Table 2B). We generated the ion channels interaction network of 141 genes (Supplementary Table 2C) comprising of 11 groups using STRING database. Interaction network shows the highly connected network of voltage-gated calcium, cation channels, voltage-gated potassium and voltage-gated sodium channels (Supplementary Figure 4A). Domain structures of nine genes highlighting the predicted deleterious somatic mutations are shown in Supplementary Figure 4B.

**Table 2:** Somatic mutations in the genes involved in ion channels in a presented case.

**Gene**	**Chr: position**	**Ref allele**	**Mut allele**	**Codon change**	**Protein change**	**Variant type**
*CACNA1S*	chr1: 201013512	C	T	c.G4741A	p.E1581K	Missense
*CACNA2D1*	chr7: 81679941	T	A	c.A883T	p.S295C	Missense
*CACNA2D4*	chr12: 1994227	C	A	c.G1084T	p.V362L	Missense
*CACNG1*	chr17: 65040987	C	A	c.C211A	p.P71T	Missense
*CACNG8*	chr19: 54486035	G	T	c.G1210T	p.G404W	Missense
*CATSPERG*	chr19: 38852337	C	T	c.C1930T	p.R644C	Missense
*GRIN2B*	chr12: 13716886	C	T	c.G3286A	p.A1096T	Missense
*KCNA2*	chr1: 111146924	G	T	c.C481A	p.P161T	Missense
*KCNC1*	chr11: 17757596	G	T	c.G47T	p.G16V	Missense
*KCNH4*	chr17: 40314304	C	T	c.G2620A	p.E874K	Missense
*KCNK4*	chr11: 64064716	C	A	c.C439A	p.R147S	Missense
*KCNK9*	chr8: 140631235	G	T	c.C391A	p.R131S	Missense
*KCNQ2*	chr20: 62103598	G	C	c.C219G	p.F73L	Missense
*P2RX7*	chr12: 121613196	C	A	c.C887A	p.A296D	Missense
SCN8A	chr12: 52115367	G	A	c.G1673A	p.R558H	Missense
*TRPM1*	chr15: 31323335	−	GTAGC	c.3028_3029insGCTAC	p.V1010fs	Frameshift insertion
*TRPM5*	chr11: 2439428	G	C	c.C875G	p.S292C	Missense
*AQP7*	chr9: 33386193	G	A	c.C407T	p.T136M	Missense

## Discussion

The incidence of UC has been rising with increased life expectancy. UC occurs mainly in older people however, young patients with UC are reported in rare cases. In this case report, we presented a study of a tumor which progressed aggressively, and the patient died on the 72 day of presentation. A typical tumor exhibits two to five driver genes (21), however our sequencing analysis of the primary tumor identified eight predicted somatic driver mutations as well as the predicted deleterious somatic mutations in genes involved in ion channels, such as *CACNA1S, KCNK9, SCN8A*, and *P2RX7*. Mostly, these somatic mutations were predicted by multiple tools (Supplementary Figure 5). A study by Biasiotta et al. have reported the significantly altered expression of *CACNAD1, CATSPER, CATSPER2, KCNN1, KCNN4, TRPM2, TRPM4, TRPV4*, and *AQP3* in the bladder carcinoma (22). Several landmark studies have been performed to study the role of ion channels in the tumorigenesis. Jacquemet et al. have reported that *CACNA1S* promotes filopodia stability and maturation in breast cancer cell lines (23). Overexpression of *KCNK9*, a proto-oncogene has been reported in breast tumors (24). Carrithers and colleagues have reported that *SCN8A* contributes to cell invasion via podosome and invadopodia formation in macrophages derived from human monocytic leukemia and melanoma cancer cells (25).

Several studies provide evidence for the role of ion channels in carcinogenesis. However, limited studies have been conducted to observe the significance of ion channels as a potential therapeutic target. For example, inhibition of *CACNA1S* has been reported to block invasion in breast cancer cell lines (23) and pancreatic cancer (26). The blocking of voltage gated potassium channels in small cell lung cancer (27), melanoma cells (28), breast cancer cells (29), and prostate cancer cells (30) with therapeutic agents have also been reported to reduce the cell proliferation. Thus, a growing body of research demonstrates that ion channels could be potential therapeutic targets for UC. Currently, the large availability of pharmacological agents targeting the majority of ion channels: amlodipine and cilnidipine, calcium channel blockers in breast cancer (26); Iberiotoxin, charybdotoxin and clotrimazole, potassium channel blockers in breast and cervical cancers (31); tetrodotoxin, voltage gated sodium channels blocker in breast cancer (32) and others, offer a broad therapeutic avenue for anticancer therapy.

## Conclusions

Our results underpin the value of WES in revealing the somatic mutations in the known cancerdriver genes and genes involved in ion channels in a patient. Ion channels could be further explored as a potential class of oncological targets for future therapeutics in advanced stage urothelial carcinoma.

## Data Availability

This manuscript contains previously unpublished data. The name of the repository and accession number are not available.

## Ethics Statement

Written informed consent was obtained from the parents of the participant for the publication of this case report. The study was approved by the ethics committee of the PGI under number PGI/IEC/2018/000874, dated: 01.06.2018.

## Author Contributions

PK conceptualized and designed the entire study. SP, AKM, and RSM carried out the sample collection from the patient. NK provided pathology images. SK and KC carried out the sequencing experiments. JS analyzed and interpreted the exome sequencing data. BD, IAG, RSM, JS and PK were involved in the preparation of the manuscript and the figures were prepared by BD and IAG.

### Conflict of Interest Statement

SK and KC are employed by MedGenome Labs Ltd. The remaining authors declare that the research was conducted in the absence of any commercial or financial relationships that could be construed as a potential conflict of interest.

## Footnotes

^1^https://www.genenames.org/data/genegroup/#!/group/177
