# Supplementary material for: Somatic Mutations Profile of a Young Patient With Metastatic Urothelial Carcinoma Reveals Mutations in Genes Involved in Ion Channels
Source: Front Oncol. 2019 May 29;9:435. doi: 10.3389/fonc.2019.00435 (PMC6549525; doi:10.3389/fonc.2019.00435)
Supplement: Supplementary file 3 [file Data_Sheet_1.docx]

Supplementary Material

**Somatic mutations profile of a young patient with metastatic urothelial carcinoma reveals mutations in genes involved in ion channels**

Jyoti Sharma, Barnali Deb, Irene A. George, Shruthi Kapil, Karunakaran Coral, Nandita Kakkar, Smita Pattanaik, Arup Kumar Mandal, Ravimohan S. Mavuduru^*^ and Prashant Kumar^*^

*** Correspondence:**

**Corresponding Author:** prashant@ibioinformatics.org

**Co-corresponding Author**: ravismi2003@yahoo.com

**Materials and Methods**

**DNA isolation, library preparation and exome sequencing:** Genomic DNA was extracted from the tumor tissue and blood samples using a Qiagen kit (QIAsymphony DNA midi Kit, Cat # 931255) using the protocols provided (Qiagen, Germantown, MD, USA). Library preparation was performed using Agilent SureSelectXT Human All Exon V5 kit as per manufacturer’s instructions. Briefly, the DNA fragments obtained from shearing were end-repaired and phosphorylated, followed by adenylation of 3' ends and ligation of standard paired end adaptors. Amplification were performed to obtain whole genome libraries. Hybridization for enriching the WG libraries for exome regions was carried out at 65°C for 16 hours using 700ng of the DNA library with addition of biotin-labeled cRNA probe sets, designed specifically for the desired targets. Dynabeads® MyOne™ Streptavidin T1 beads (Catalog # 65602, Thermo Fisher Scientific) were used for capture of resulting DNA-cRNA duplexes. Multiple washes at high stringency were performed to remove any bound off-target material and any non-hybridized fragments. Specific libraries were then amplified using indexed primers and Herculase II Fusion DNA Polymerase (Catalog #600675, Agilent Technologies Inc.). Subsequently, cluster amplification was performed according to manufacturer’s protocol (Illumina ,USA) using Illumina platform with read length of 150X2 bp.

**Whole‑exome sequencing data analysis:** Quality analysis and processing of the raw reads was carried out using FastQC (v0.11.5) and Trimmomatic ([1](#_ENREF_1)) tools, respectively. Quality processed paired‑end reads were aligned to the to the human reference genome hg19/GRCh37 using BWA-MEM algorithm ([2](#_ENREF_2)) with default parameters. Picard tool ([3](#_ENREF_3)) was used for post-alignment procedures including sorting and indexing. The alignments were submitted to marking duplicates and base quality score recalibration (BQSR) by using the Genome Analysis Toolkit (GATK) version . 4.0.2.1 ([4](#_ENREF_4)). Somatic mutations were called using Mutect2 ([5](#_ENREF_5)). The identified somatic mutation candidates were annotated using ANNOVAR tool ([6](#_ENREF_6)). Also, somatic mutations were called using Strelka2 ([7](#_ENREF_7)) and Varscan 2 ([8](#_ENREF_8)). Interaction network of mutated genes containing somatic mutations was generated using STRING database ([9](#_ENREF_9)).

**References:**

1. Bolger AM, Lohse M, Usadel B. Trimmomatic: a flexible trimmer for Illumina sequence data. *Bioinformatics* (2014) 30(15):2114-20. Epub 2014/04/04. doi: 10.1093/bioinformatics/btu170. PubMed PMID: 24695404; PubMed Central PMCID: PMC4103590.

2. Li H, Durbin R. Fast and accurate long-read alignment with Burrows-Wheeler transform. *Bioinformatics* (2010) 26(5):589-95. Epub 2010/01/19. doi: 10.1093/bioinformatics/btp698. PubMed PMID: 20080505; PubMed Central PMCID: PMC2828108.

3. http://broadinstitute.github.io/picard/

4. Van der Auwera GA, Carneiro MO, Hartl C, Poplin R, Del Angel G, Levy-Moonshine A, et al. From FastQ data to high confidence variant calls: the Genome Analysis Toolkit best practices pipeline. *Current protocols in bioinformatics* (2013) 43:11 0 1-33. Epub 2014/11/29. doi: 10.1002/0471250953.bi1110s43. PubMed PMID: 25431634; PubMed Central PMCID: PMC4243306.

5. do Valle IF, Giampieri E, Simonetti G, Padella A, Manfrini M, Ferrari A, et al. Optimized pipeline of MuTect and GATK tools to improve the detection of somatic single nucleotide polymorphisms in whole-exome sequencing data. *BMC bioinformatics* (2016) 17(Suppl 12):341. Epub 2017/02/12. doi: 10.1186/s12859-016-1190-7. PubMed PMID: 28185561; PubMed Central PMCID: PMC5123378.

6. Wang K, Li M, Hakonarson H. ANNOVAR: functional annotation of genetic variants from high-throughput sequencing data. *Nucleic acids research* (2010) 38(16):e164. Epub 2010/07/06. doi: 10.1093/nar/gkq603. PubMed PMID: 20601685; PubMed Central PMCID: PMC2938201.

7. Kim S, Scheffler K, Halpern AL, Bekritsky MA, Noh E, Kallberg M, et al. Strelka2: fast and accurate calling of germline and somatic variants. *Nature methods* (2018) 15(8):591-4. Epub 2018/07/18. doi: 10.1038/s41592-018-0051-x. PubMed PMID: 30013048.

8. Koboldt DC, Zhang Q, Larson DE, Shen D, McLellan MD, Lin L, et al. VarScan 2: somatic mutation and copy number alteration discovery in cancer by exome sequencing. *Genome research* (2012) 22(3):568-76. Epub 2012/02/04. doi: 10.1101/gr.129684.111. PubMed PMID: 22300766; PubMed Central PMCID: PMC3290792.

9. Szklarczyk D, Morris JH, Cook H, Kuhn M, Wyder S, Simonovic M, et al. The STRING database in 2017: quality-controlled protein-protein association networks, made broadly accessible. *Nucleic acids research* (2017) 45(D1):D362-D8. Epub 2016/12/08. doi: 10.1093/nar/gkw937. PubMed PMID: 27924014; PubMed Central PMCID: PMC5210637.

**Legends for supplementary figures and tables**

**Supplementary figure 1:** Proportion of different types of somatic mutations namely, missense, frame-shift insertion, frame-shift deletion, nonsense and splice site in a case presented.

**Supplementary figure 2:** The functional domain structures of eight predicted somatic driver mutations in a case presented. The missense and truncating mutations in each position are indicated with green and black circles, respectively.

**Supplementary figure 3:** The connected landscape of nonsense somatic mutations in a urothelial carcinoma. Genes harboring nonsense somatic mutations were crossreferenced against the STRING protein interaction database. Highly connected ion channels network is indicated with red circle.

**Supplementary figure 4: 1A-**The interaction network of ion channels. 141 ion channels genes belonging to 11 groups of ion channels were crossreferenced against the STRING protein interaction database. Genes harboring nonsense and frame shift insertion somatic mutation(s) are indicated in red and grey circles. **1B-** The functional domain structures of nine predicted deleterious nonsense mutations in ion channel genes. The mutations in each position are indicated with green circle.

**Supplementary figure 5:** Depiction of seventeen somatic mutations in known/predicted cancer driver and ion channels genes predicted by MuTect2, Strelka2 and VarScan 2 tools. Grey and white boxes are indicated with identified and not identified mutations, respectively.

**Supplementary table 1:** List of 558 exonic somatic mutations called and annotated using MuTect2 and ANNOVAR, respectively in a case presented.

**Supplementary table 2:** List of ion channels genes. 2A- List of 328 ion channel genes approved by HGNC. 2B- List of 11 ion channels group name harboring the 22 altered somatic mutations. 2C- List of 141 genes comprising of 11 ion channels groups.
