# Supplementary material for: Somatic Mutations Profile of a Young Patient With Metastatic Urothelial Carcinoma Reveals Mutations in Genes Involved in Ion Channels
Source: Front Oncol. 2019 May 29;9:435. doi: 10.3389/fonc.2019.00435 (PMC6549525; doi:10.3389/fonc.2019.00435)

Supplementary figure 1

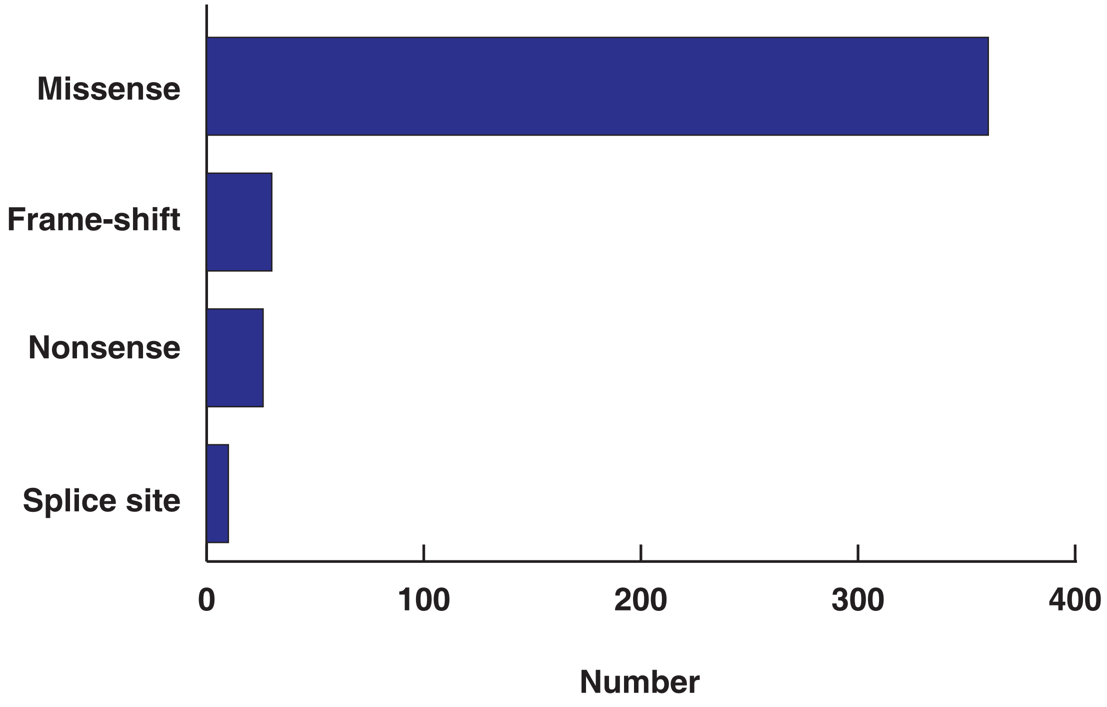

Supplementary figure 2

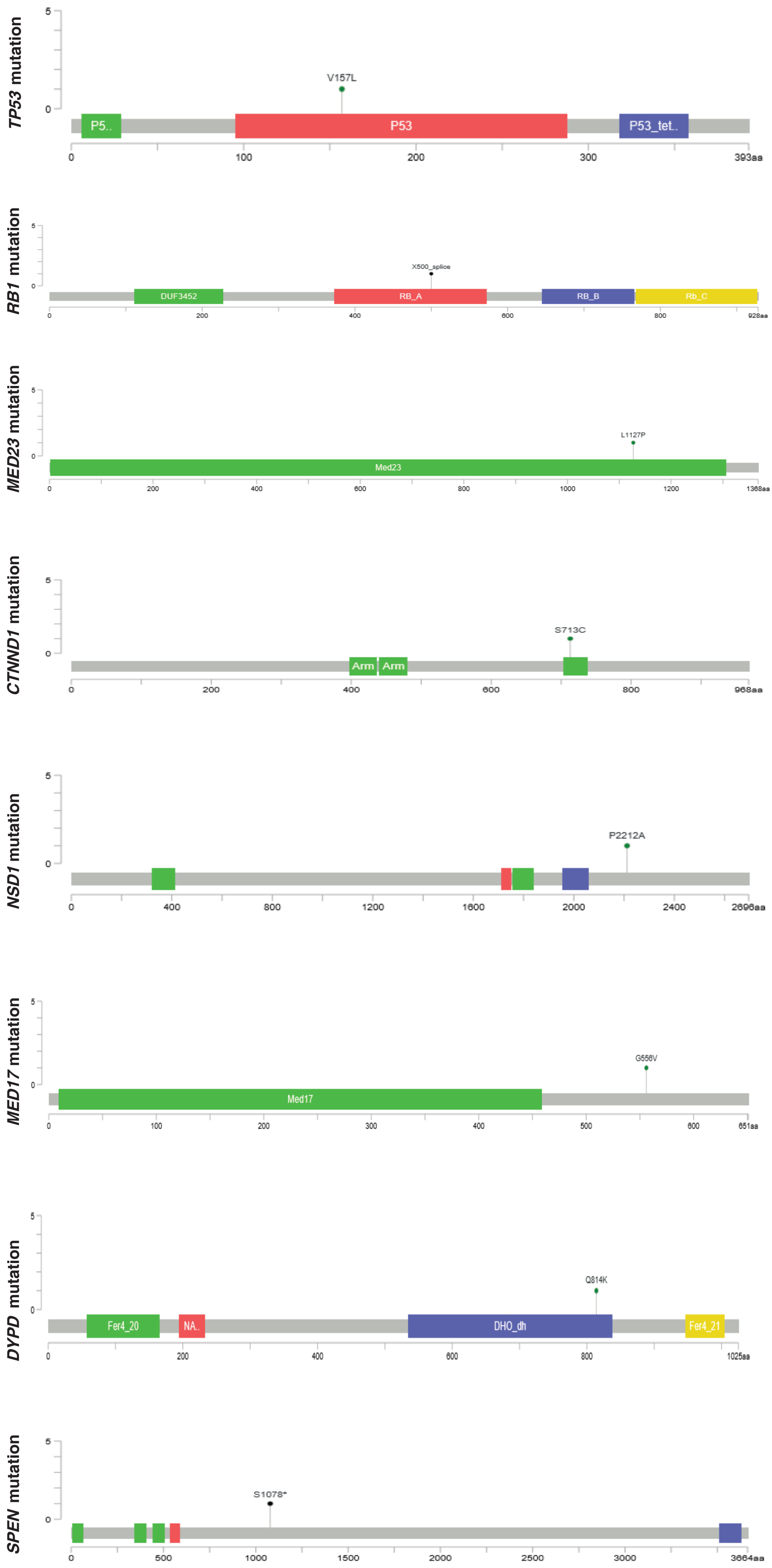

Supplementary figure 3

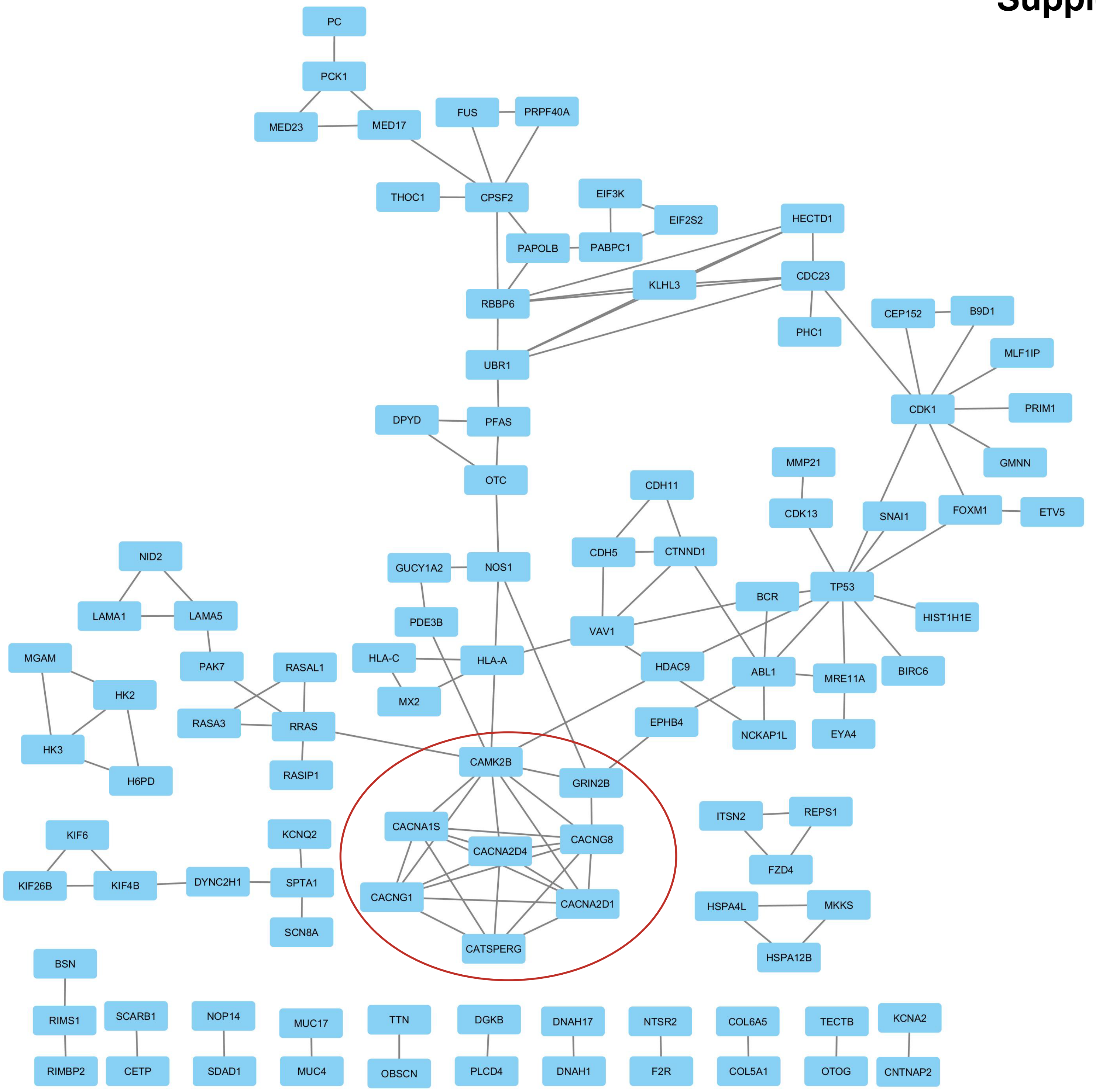

Supplementary figure 4

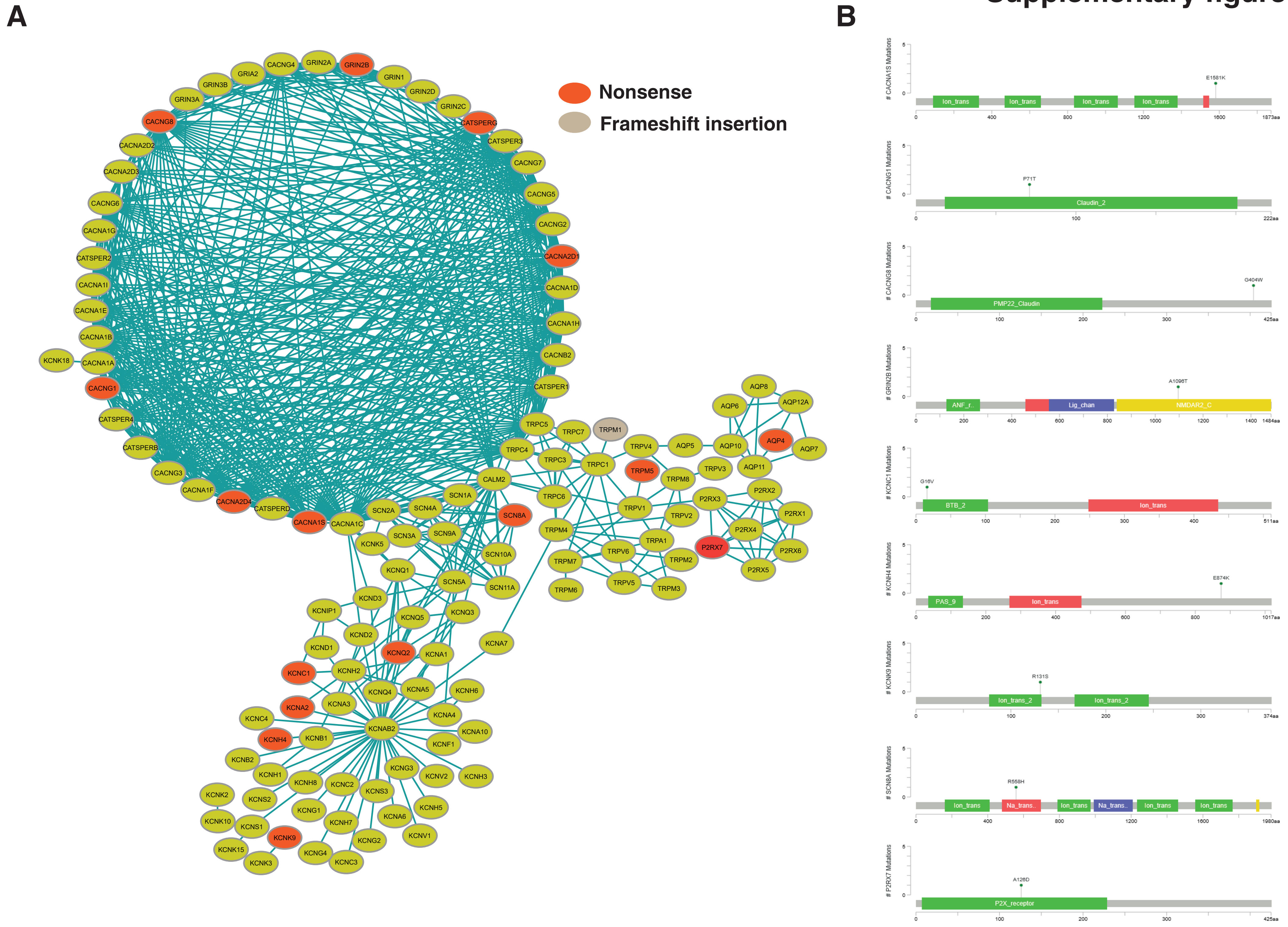

Supplementary figure 5

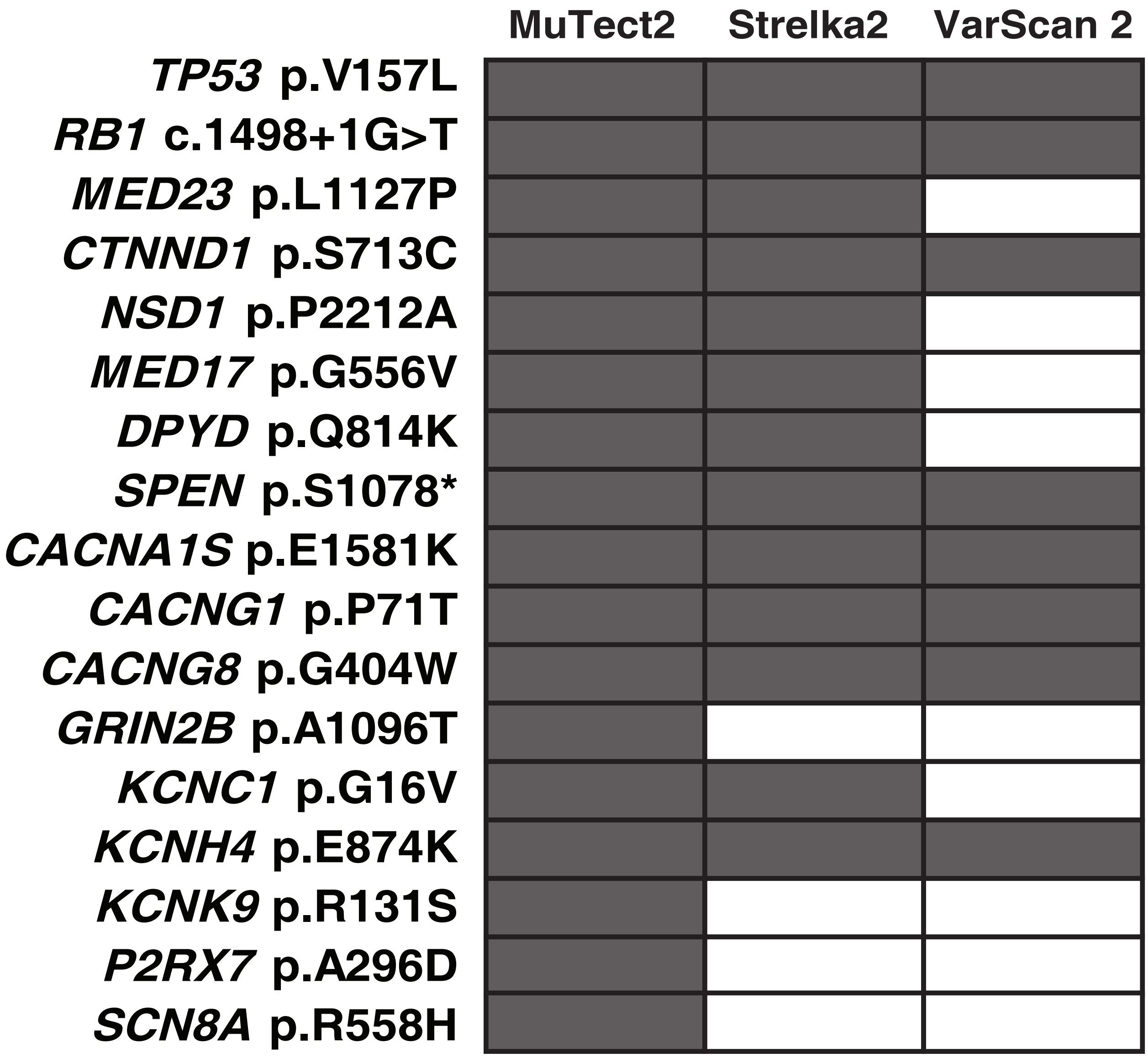

Supplement: Supplementary file 4 [file Data_Sheet_2.PDF]
